# Neighbored-attention U-net (NAU-net) for diabetic retinopathy image segmentation

**DOI:** 10.3389/fmed.2023.1309795

**Published:** 2023-12-07

**Authors:** Tingting Zhao, Yawen Guan, Dan Tu, Lixia Yuan, Guangtao Lu

**Affiliations:** ^1^The Second Department of Internal Medicine, Donghu Hospital of Wuhan, Wuhan, China; ^2^The Department of Ophthalmology, Donghu Hospital of Wuhan, Wuhan, China; ^3^Precision Manufacturing Institute, Wuhan University of Science and Technology, Wuhan, China

**Keywords:** image semantic segmentation, deep learning, diabetic retinopathy, neighbored-attention U-net, fundus image

## Abstract

**Background:**

Diabetic retinopathy-related (DR-related) diseases are posing an increasing threat to eye health as the number of patients with diabetes mellitus that are young increases significantly. The automatic diagnosis of DR-related diseases has benefited from the rapid development of image semantic segmentation and other deep learning technology.

**Methods:**

Inspired by the architecture of U-Net family, a neighbored attention U-Net (NAU-Net) is designed to balance the identification performance and computational cost for DR fundus image segmentation. In the new network, only the neighboring high- and low-dimensional feature maps of the encoder and decoder are fused by using four attention gates. With the help of this improvement, the common target features in the high-dimensional feature maps of encoder are enhanced, and they are also fused with the low-dimensional feature map of decoder. Moreover, this network fuses only neighboring layers and does not include the inner layers commonly used in U-Net++. Consequently, the proposed network incurs a better identification performance with a lower computational cost.

**Results:**

The experimental results of three open datasets of DR fundus images, including DRIVE, HRF, and CHASEDB, indicate that the NAU-Net outperforms FCN, SegNet, attention U-Net, and U-Net++ in terms of Dice score, IoU, accuracy, and precision, while its computation cost is between attention U-Net and U-Net++.

**Conclusion:**

The proposed NAU-Net exhibits better performance at a relatively low computational cost and provides an efficient novel approach for DR fundus image segmentation and a new automatic tool for DR-related eye disease diagnosis.

## 1 Introduction

Recently, as the number of patients with diabetes mellitus (DM) has increased greatly and they tend to be younger, an increasing number of people suffer from diabetic retinopathy (DR) (1, 2). As an eye disease, DR may cause visual impairment or even blindness if not diagnosed and treated in a timely manner (3). DR typically results in optic disc (OD) lesions. These lesions involve abnormal changes in retinal blood flow, and the abnormalities primarily include microaneurysms (MA), hard exudates, soft exudates, hemorrhages (HA), neovascularization (NV), and macular edema (ME) (4). These changes in the OD can be captured and recorded in images using a DR screening device, and OD abnormalities can be easily distinguished by experienced doctors by analyzing the fundus images. However, the manual diagnosis of DR requires doctors to check numerous images, which is time-consuming, resource-intensive, and expensive.

Owing to the increasing development of computer vision technologies, deep learning methods, especially image identification technology including image classification and image semantic segmentation method, have been introduced for automatic diagnosis of DR. As a method of image identification technologies based on computer vision, image classification algorithms typically preprocess the images first using image processing technologies and then enhance or extract some features from the preprocessed images, including histograms of oriented gradients (HOG), higher-order spectra (HOS), and speeded-up robust features (SURF). The extracted features are input into an intelligent classifier model with a category or label. After the classifier is trained, it is used to predict a new DR fundus or other medical images. It outputs a category or label that represents the type of disease (5, 6). The commonly used classifiers include support vector machine (SVM), genetic algorithm (GA), and convolutional neural networks (CNN) (4, 7). Orfao and Haar (8) compared the performance of different classifiers, and their experimental results indicated that the radial basis function SVM (RBF-SVM) model obtained a higher accuracy and F1-score using the HOG feature of the green channel. Ghoushchi et al. (9) combined fuzzy C-mean (FCM) and GA algorithms to identify diabetic and nondiabetic eye images with a relatively high recognition rate. Li et al. (10) obtained the features of the DR1 and Messidor datasets using a fine-tuning CNN and used an SVM model to classify the images. Le et al. (11) first selected the feature using an adaptive particle-grey wolf optimization method and classified the image using a multilayer perceptron (MLP). Their comparative results showed that the new algorithm predicted the images with a higher accuracy.

Moreover, because the CNN model shows a powerful ability for image enhancement, various CNN models have been introduced into image feature selection. CNN models are typically connected by a series of convolutional, activation, pooling, dropping, and fully connected layers, and based on the architecture of the backbones of the CNN, various CNN models, including AlexNet, VGG, DenseNet, ResNet, MobileNet, are used for DR and other medical image segmentation. Shanthi and Sabeenian (12) used an AlexNet with four convolution layers and three pooling layers to augment the fundus images of the Messidor dataset and classified the severity using filtered data. Khan et al. (13) modified the architecture of VGG16 to improve the performance of DR image diagnosis and tested the identification performance using the Kaggle dataset. Kobat et al. (14) first separated the DR image into parts by resizing and dividing the original image and then trained DenseNet201 and SVM classifiers to augment and estimate the DR images, respectively. Al-Moosawi and Khudeyer (15) diagnosed four different categories of DR using a trained ResNet34 and compared the performances of different DL architectures. The identification results of the fundus images from APTOS 2019 and IDRiD showed that ResNet34 performed better in image feature enhancement. Moreover, considering its powerful target detection ability, the popular Yolo V3 model was introduced for automatic DR fundus image identification by Pal et al. (16). Similar studies have been conducted by Wang et al. (17), Das et al. (18), Mohamed et al. (19), and Santos et al. (20).

In contrast to the aforementioned image classification methods, image semantic segmentation methods detect and classify images at each pixel (21, 22). Therefore, after semantic image segmentation, the retinal blood vessels or other important structures of the DR or other medical images are augmented, and the lesion area is directly detected and located. Image semantic segmentation algorithms are derived from or based on CNN, and typical image semantic segmentation architectures are fully convolutional networks (FCN), SegNet, pyramid scene parsing networks (PSPNet), DeepLab, Unet, etc. (23, 24). To achieve a tradeoff between semantic and location information, Wang et al. (25) improved the original R-FCN by adding an upsampling unit in the common ResNet101 and used a feature pyramid network to generate a feature map with different feature map levels. Using the modified R-FCN, higher sensitivity and specificity for DR image segmentation were obtained. To increase the feature map resolution, the original SegNet used an encoder to obtain the feature maps and employed a decoder to up-sample the feature maps (26). SegNet was first proposed by Saha et al. (27) for road and indoor scene segmentation, and Ananda et al. (28) introduced SegNet for DR image segmentation. To make optimal use of the global feature in image segmentation tasks, a global pyramid pooling layer and certain new strategies were proposed in PSPNet and compared with FCN (29). Fang et al. (30) combined a phase-up-sampling module and PSPNet for fundus image segmentation. This improved model obtained higher intersection over union (IoU) and pixel accuracy than the native PSPNet. Chen et al. (31) introduced arouse convolution and a conditional random field (32) to strengthen the boundary details and finally obtained a better image segmentation effect. This architecture is known as DeepLab v1. To further improve the identification accuracy of the boundary, DeepLab v2 (33), DeepLab v3 (34), and DeepLab v3+ (35) were developed by modifying certain modules of the DeepLab v1 network. Some researchers have reviewed and compared the performances of other networks (36).

However, the performance of these image segmentation algorithms is affected by the number of training samples. In addition, datasets of medical images, particularly images of rare cases, are typically insufficient. Therefore, the U-Net was first reported by Ronneberger et al. (37) to improve the performance of small-sample image segmentation. U-Net uses a symmetric architecture to suppress the key image features by down-sampling and to extract low-level features by skip connection and up-sampling. It finally exhibits excellent performance by fusing all the features. Moreover, various variants of U-Net have been developed by modifying or adding modules to improve their accuracy. However, these variants typically achieve excellent performance by fusing multi-scale feature maps with dense links between the encoder and decoder, and as a result, they usually need the expense of computational and time costs. Therefore, to balance the identification performance and computational of the algorithm, a novel U-Net named neighboring attention U-Net is designed for DR fundus image semantic segmentation.

The paper is structured as follows: Section 2 summarizes and discusses the studies on U-Net and its variants. Section 3 introduces the architecture and workflow of the proposed network. Section 4 provides the details of the datasets and compares the testing performances of the different networks. Section 5 summarizes the whole study.

## 2 Related previous works of U-Net family

**Figure 1:**
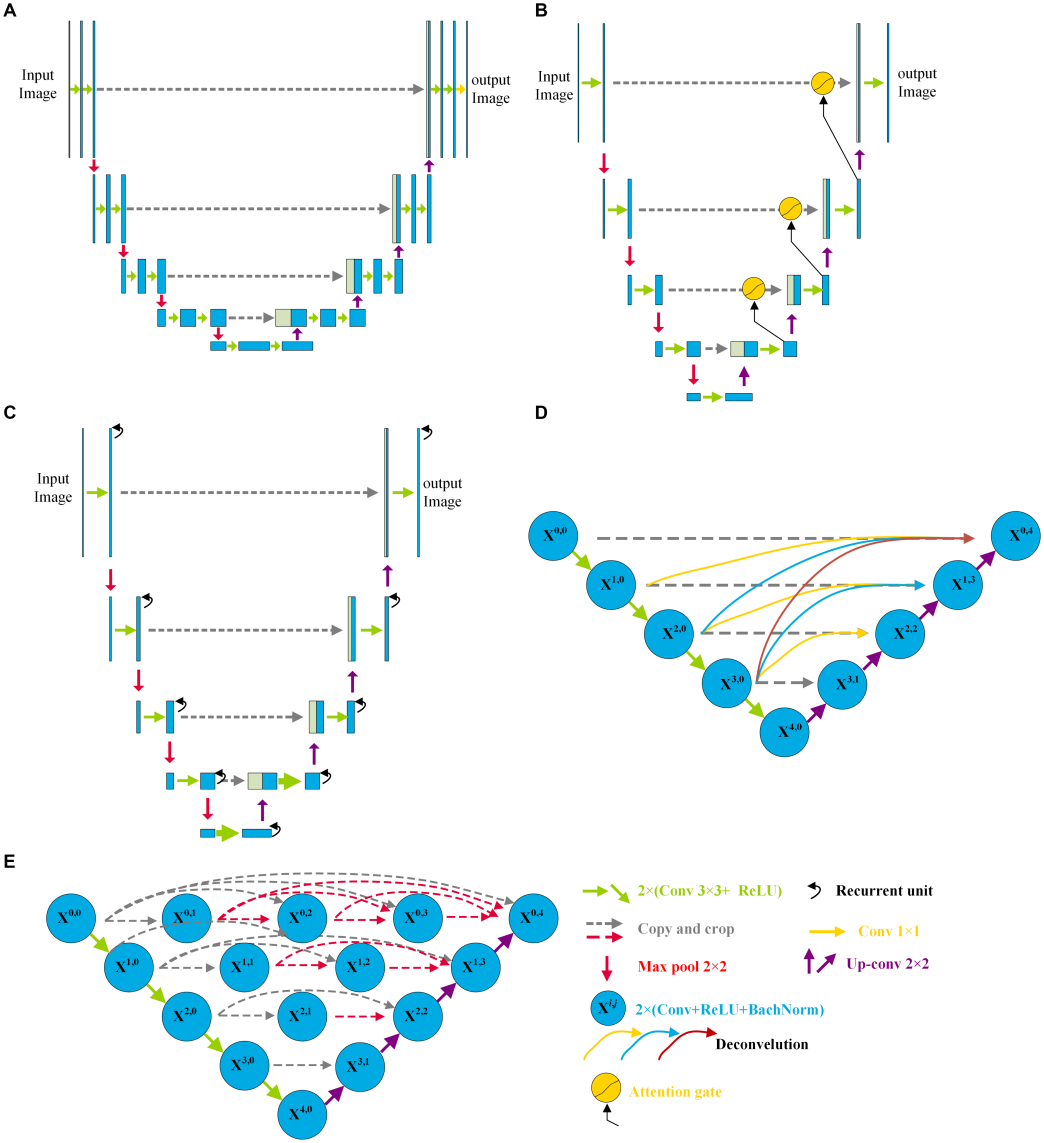
Architectures of some variants of U-Net: **(A)** U-Net, **(B)** Attention U-Net, **(C)** R2U-Net, and **(D)** CE-Net; **(E)** U-Net++.

Since the U-Net was first reported by Ronneberger et al. (37) in 2015, various variants of the U-Net have been developed and have displayed a wide and strong applicability for DR fundus, cell, lung, skin cancer, colorectal adenocarcinoma gland, and coronary artery image segmentation in the field of medicine. Figure 1 shows the structure of U-Net and its variants. Apart from the original U-Net, the U-Net family primarily includes attention U-Net, residual U-Net, residual-attention U-Net, recurrent residual convolutional neural network (RRCN) based on U-Net (R2U-Net), U-Net++, Nested U-Net, etc. As shown in Figure 1, in these variants, some modules are modified or added to further focus on their ability for image feature extraction and fusion at different levels.

Inspired by the concept of FCN, a new network with an encoder and decoder was designed in 2015, and it was called “U-Net” because of its symmetric architecture. As shown in Figure 1A, the U-Net encoder primarily consists of convolution, ReLU activation, and max pooling modules, whereas the decoder primarily consists of up-convolution, convolution, ReLU activation, and max pooling modules. Moreover, to join the features of the encoder, a cropping operation is performed at the corresponding levels in the decoding process. Owing to its innovation in image feature extraction and fusion at different levels, U-Net has displayed outstanding performance in medical image segmentation with a small sample size. Çiçek et al. (38) transformed a 2D U-Net model into a 3D U-Net for volumetric segmentation of biomedical images using 3D modules. To be more sensitive to the local region, Oktay et al. (39) added three attention gates before the copy and cropping operations in attention U-Net as shown in Figure 1B. The attention U-Net had a higher Dice score and a lower surface distance in the CT abdominal image segmentation. To simplify training and decrease the degradation of the U-Net model, Zhang et al. (40) introduced a residual mechanism into the architecture and designed a deep residual U-Net model for road image segmentation. The residual U-Net inherits the depth of the residual network and feature fusion ability at different levels. Combining the advantages of the residual, recurrent, and U-Net modules, Alom et al. (41) designed R2U-Net in 2019. In R2U-Net of Figure 1C, the introduction of RRCN modules further enhances the feature extraction ability at each pixel and increases its depth. Owing to the powerful abilities of the modules, R2U-Net displayed a better response than U-Net in various medical image segmentations. Considering that the pooling and convolution operations of U-Net typically result in a loss of feature resolution and spatial information, Gu et al. (42) designed a network called CE-Net shown in Figure 1D, based on U-Net. In addition to the encoder and decoder, CE-Net has a context extractor for dense atrous convolution and residual modules. The advantages of the proposed context extractor in CE-Net are compared and proven by segmenting different types of images. Moreover, to achieve high accuracy in medical image segmentation, Zhou et al. (43) nested different layers of U-Net by adding new skip pathways; therefore, this network is called U-Net++ or Nested U-Net. As shown in Figure 1E, in U-Net ++, the redesigned pathways mapped the feature maps of the encoder to the decoder; consequently, the feature maps of the two networks were fused. As the number of pathways increased significantly, the parameters of the model expanded, and the computational cost increased. The experimental test of CT image segmentation showed that it achieved an average IoU improvement of approximately 3%, and its total parameters increased by approximately 16.5% compared with U-Net. To balance the computational cost and segmentation performance, the AdaBoosted supervision mechanism was added to U-Net, and this architecture was called ADS_U-Net (44). In this model, deep supervision and performance-weighted combination were conducted to reduce the correlations between different feature maps and obtain excellent comprehensive performance in image segmentation and computation costs. Inspired by U-Net++, Li et al. (45) proposed a residual-attention U-Net++ in which the residual and attention modules were embedded into U-Net++. With the assistance of these two modules, the degradation was weakened and irrelevant features were filtered; therefore, the target feature was enhanced. As a result, the modified U-Net++ obtained higher IoU and Dice scores.

As shown in Figure 1, compared with the original architecture of U-Net, attention U-Net, U-Net++, and residual-attention U-Net++ have more links between the low- and high-dimensional feature maps, and these features of different levels are well combined, which filters the low-relevance features and boosts the target features. More complicated nested layers assist in improving the performance; however, they introduce a larger number of parameters and increase the computational cost. Therefore, to balance computational performance and cost, neighbored attention U-Net (NAU-Net) is proposed for DR and other medical image segmentation. In this new network, neighboring high- and low-dimensional feature maps are fused by an attention gate to filter the target features at a relatively low cost.

## 3 Methodology

### 3.1 Whole architecture of NAU-Net

**Figure 2:**
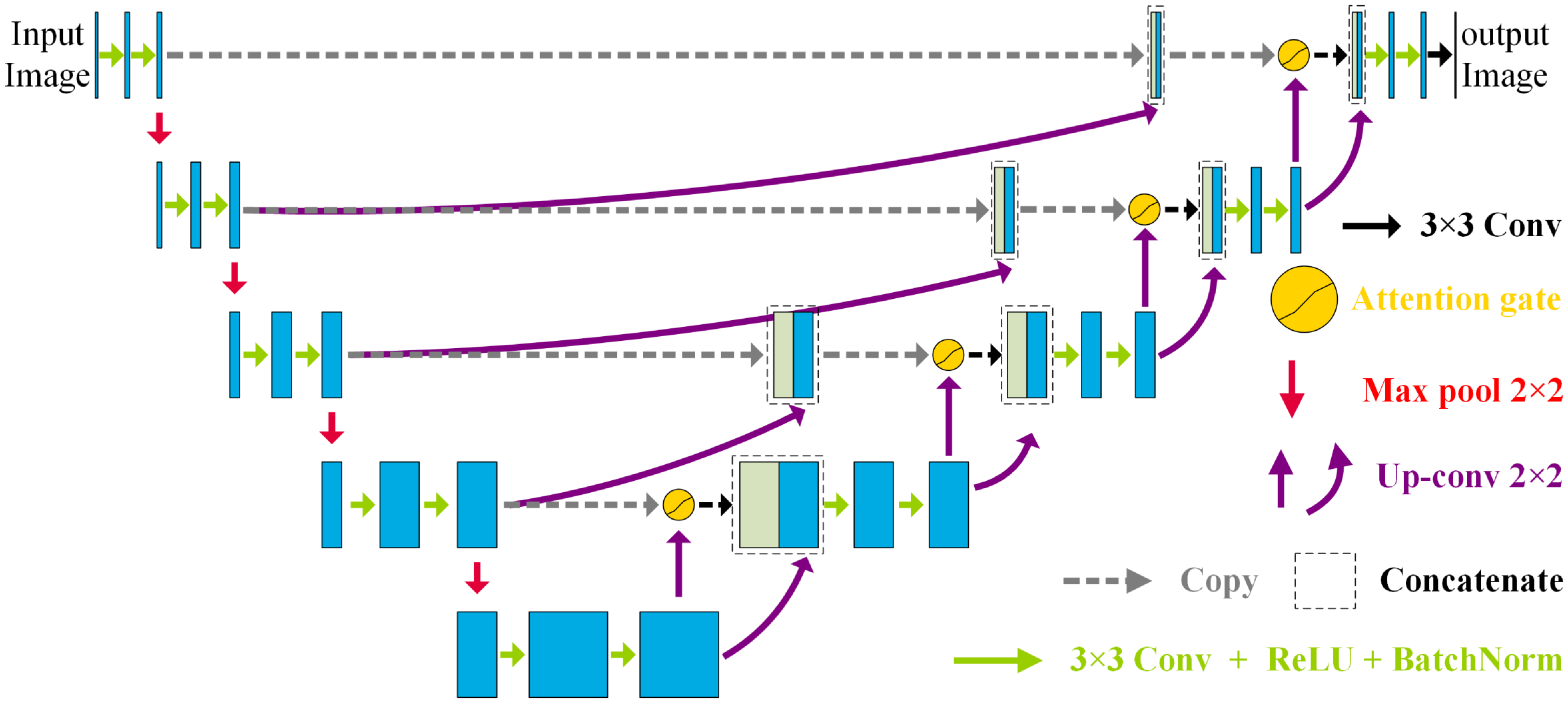
Structure of the NAU-Net.

Figure 2 shows the NAU-Net’s structure. As shown in Figure 2, the NAU-Net adds four attention gates to map the feature maps of the encoder to the decoder at different levels. The inputs to the attention gate are the two neighboring feature maps of the encoder and decoder at the same level. Using these attention gates, similar feature maps are fused, and the target features are enhanced. Moreover, this network only uses neighboring layers and does not include the inner layers commonly used in U-Net++ and residual attention U-Net++. Consequently, the proposed network incurs a lower computational cost.

To fuse the feature maps conveniently and make the output size similar to the input image, the conventional kernel size is 3 × 3, and its stride and padding are one. After the convolution operation, the ReLU, batch normalization, and max pooling operations are performed. The maximum pooling is 2 × 2, and the stride is two. The up-convolution operation included up-sampling, 2 × 2 convolution with a stride and padding of one, batch normalization, and ReLU operations. Finally, a 3 × 3 convolution operation transfers the filtered image to one channel.

### 3.2 Neighbored feature maps fusion

As the convolutional layers of encoder increase, more and more detailed features of the target get loss. However, there is some similarity between the two-neighboring high-dimensional feature maps in the encoder, and this connection between the maps faraway gets weaker. Therefore, to enhance the common features in the maps with a relative low computation cost, only the two neighboring feature maps of the encoder are fused by an attention in NAU-Net.

**Figure 3:**
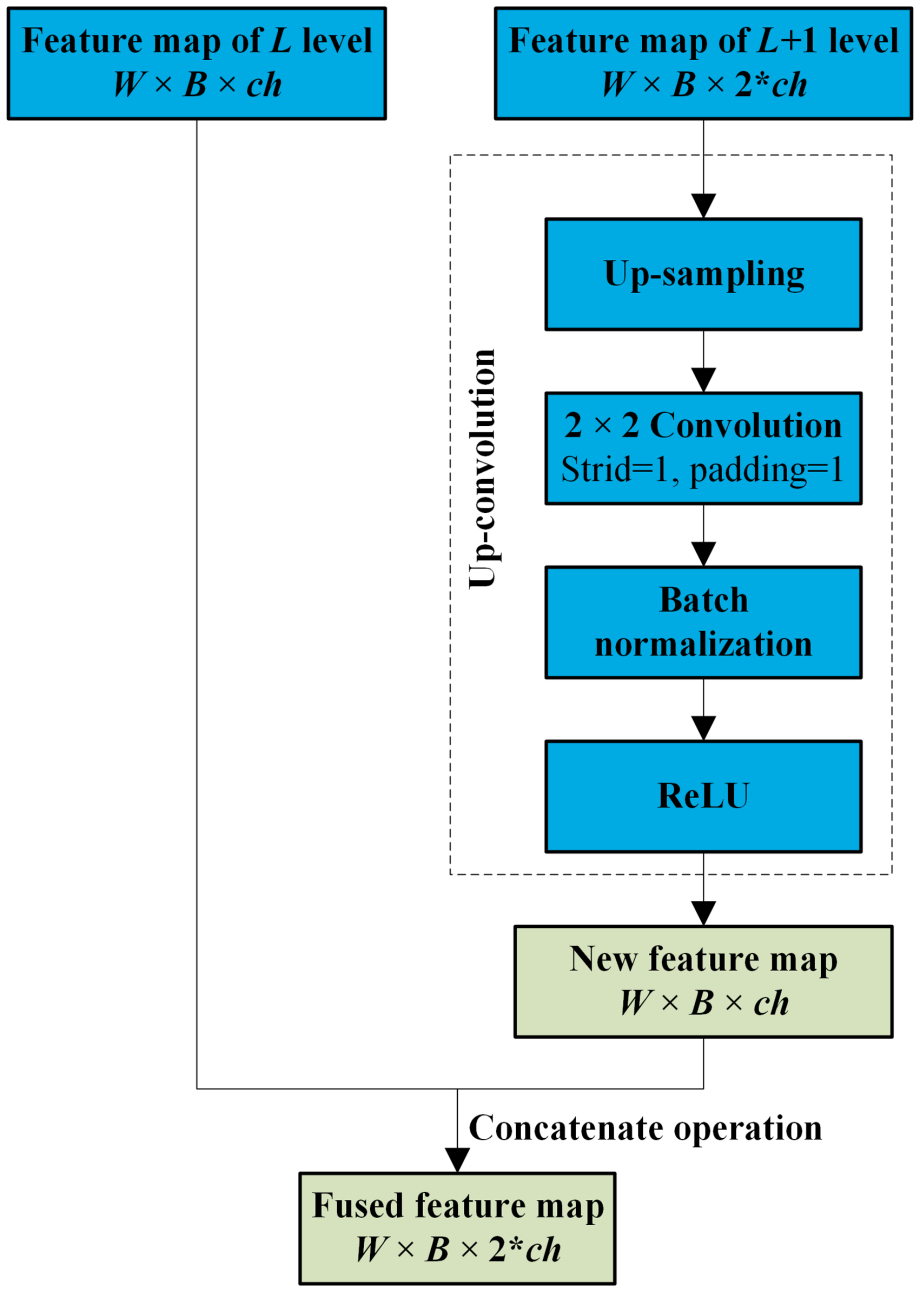
Neighbored feature maps fusion.

Before the feature maps of the encoder and decoder are combined, the two neighboring feature maps of the encoder are fused. Because the dimensions of the two neighbored feature maps of the encoder are different, the lower-dimensional feature map is first filtered by an up-convolution operation and then fused by a concatenation operation. The entire fusion operation is shown in Figure 3. As shown in Figure 3, after the feature map of the L + 1 level with dimensions *W* × *B* × 2 × *ch* is processed by up-sampling, convolution, batch normalization, and ReLU sequentially, a new feature map with the same dimensions as the *L*th feature map is obtained. Subsequently, the new feature map is concatenated with the *L*th feature map.

### 3.3 Attention mechanism in NAU-Net

**Figure 4:**
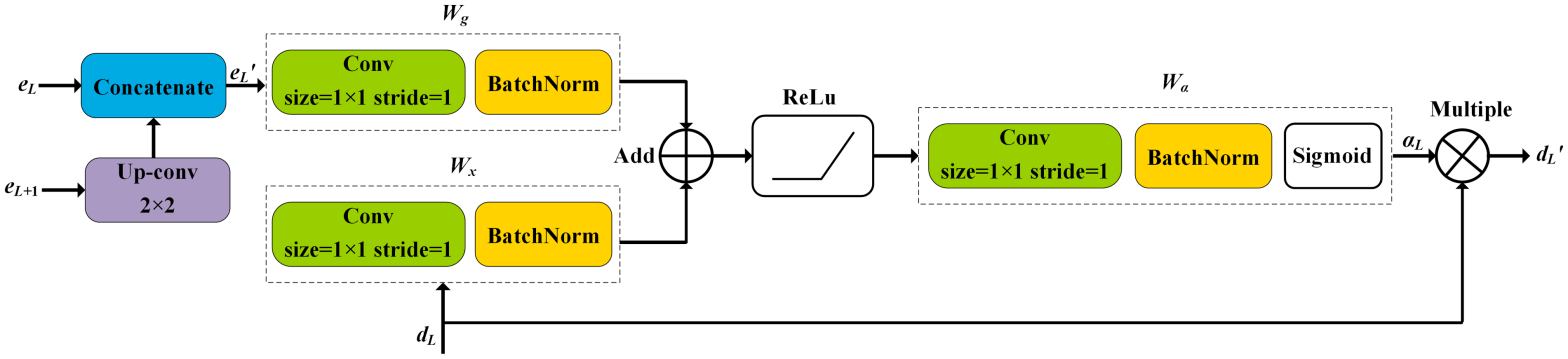
Attention gate in NAU-Net.


The high-dimensional feature maps of the encoder usually contain fine-grained features of the target, while the low-dimensional ones of the decoder contain coarse texture of the target. Therefore, to increase the identification accuracy, the multi-scale features of the target in low- and high-dimensional feature maps are extracted and fused by the attention mechanism in NAU-Net. Figure 4 shows the entire procedure for the attention mechanism in NAU-Net. In Figure 4, the low- and high-dimensional feature maps are inputted to a common attention gate, and the output $d_{L}^{\prime }$ of the attention gate is expressed as follows:

(1) $$q_{L}=\sigma_{1}\left(w_{g}\left(e_{L}^{\prime }\right)+w_{x}\left(d_{L}\right)\right)$$

(2) $$\alpha_{L}=w_{\alpha }\left(q_{L}\right)$$

(3) $$d_{L}^{\prime }=\alpha_{L}d_{L}$$

where *σ*_1_ represents the ReLU operation, *d_L_* represents the feature map of decoder at the level *L*, *w_g_* and *w_x_* represent the plain convolution and batch normalization operations of the feature maps $e_{L}^{\prime }$ and *d_L_,* respectively, *α_L_* represents the attention coefficient, *w_α_* represents the combining operation convolution, batch normal, and sigmoid activation. It is noteworthy that the kernel size of the attention gate convolution is 3 × 3 with a stride of 1.

### 3.4 Loss function

In this study, binary cross-entropy and Dice loss (BCE-Dice loss) are selected as loss functions to evaluate segmentation performance (46). The *i*th predicted image and its corresponding ground-truth image are *p_i_* and *g_i_*. The BCE-Dice loss is expressed as follows:


(4) $$\begin{array}{l} L_{BCE-\mathrm{Dice}}=-\frac{1}{N}\overset{N}{\underset{i=1}{\sum }}\left(p_{i}\log\left(g_{i}\right)+\left(1-p_{i}\right)\log\left(1-g_{i}\right)\right)\\ \;+\frac{1}{N}\overset{N}{\underset{i=1}{\sum }}\left(1-\frac{2TP_{i}}{2TP_{i}+FP_{i}+FN_{i}}\right) \end{array}$$

where *N* is the total number of images and *TP_i_*, *FP_i_*, and *FN_i_* are the true positives, false positives, and false negatives of the *i*th predicted image, respectively.

## 4 Experiments and results

**Figure 5:**
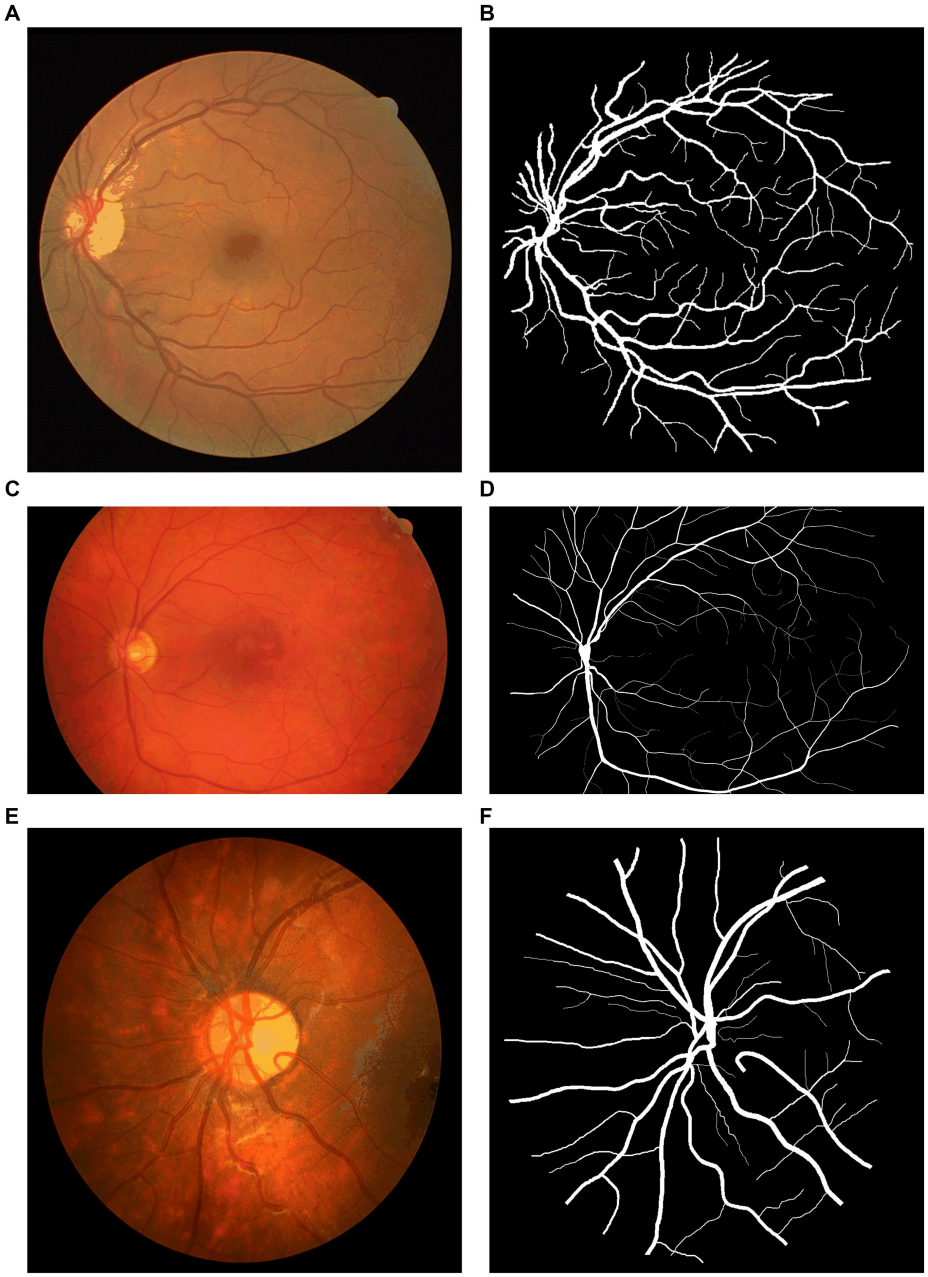
Fundus images of open datasets: **(A)** DRIVE, **(B)** Ground true image of **(A,C)** HRF; **(D)** Ground truth image of **(C)**, **(E)** CHASEDB, and **(F)** Ground truth image of **(E)**.

To test and compare the performance of NAU-Net, datasets of DR fundus images, including digital retinal images for vessel extraction (DRIVE), high-resolution fundus (HRF), and CHASEDB, were tested. Figure 5 shows the fundus images from the three datasets. Moreover, the segmentation performance of NAU-Net was compared with FCN, SegNet and two variants of U-Net, namely attention U-Net and U-Net++, whose networks are similar to the proposed model. The proposed model and a few existing networks were established by using the PyTorch framework (version 1.10.0), and all experimental tests were conducted at the High-Performance Computing Center at Wuhan University of Science and Technology. All the tests were conducted on a computer with four NVIDIA Tesla V100S GPUs, and the memory capacity of each GPU board was 32 GB.

### 4.1 Datasets

#### 4.1.1 DRIVE dataset

The total of 40 color DR fundus images from DRIVE were used in this study (47). The resolution of the images was 584 × 565 pixels per channel, and each image had three channels. The ratio of the training and testing split was 20:20. The ground truth of each image was manually segmented and marked by one or two different ophthalmological experts.

#### 4.1.2 HRF dataset

The HRF (48) included 45 original DR fundus images, including 15 healthy, 15 DR, and 15 glaucomatous fundus images. All images were manually marked by experts. The image resolution was 3,504 × 2,336 pixels. Moreover, in this study, healthy and DR fundus images were imported; among them, 26 images were selected as the training set, and the remaining four were selected as the testing set.

#### 4.1.3 CHASEDB dataset

The 28 color fundus images from CHASEDB (49) were also used to display the performance of NAU-Net. Each image contained 999 × 960 pixels and was marked by two independent experts. The training and testing sets contained 21 and seven images, respectively.

#### 4.1.4 Evaluation metrics

To display and compare segmentation performance, some commonly used evaluation metrics, including the Dice score, IoU, accuracy (AC), and precision (PC), were introduced in this study. These four metrics are obtained as follows:


(5) $$DC=\frac{2TP}{FP+FN+2TP}$$

(6) $$IoU=\frac{TP}{FP+FN+TP}$$

(7) $$\mathrm{AC}=\frac{TP+TN}{TP+TN+FP+FN}$$

(8) $$PC=\frac{TP}{TP+FP}$$

where *TP*, *TN*, *FP,* and *FN* represent the true positives, true negatives, false positives, and false negatives, respectively.

Moreover, the computational cost was evaluated by comparing the total number of parameters and GPU memory demands of the models.

### 4.2 Results

During the inference process, the Adam optimizer was selected, and its learning rate was adjusted using the CosineAnnealinLR scheduler. The maximum number of iterations was 10. The minimum learning rate of the scheduler was 0.0001. The total number of epochs was 140, and the batch size was selected as four. All images were resized to 576 × 576 pixels before inference. Before the images were put into the model, they were preprocessed by normalization with parameters mean = [0.485, 0.456, 0.406] and std. = [0.229, 0.224, 0.225]. Moreover, the information of libraries used in this study is available at website https://github.com/Aynor007/MyNAUnet.

#### 4.2.1 Computation cost comparison

To evaluate the computational cost of NAU-Net, the number of parameters, total memory demand, and complexity of different models, including FCN, SegNet, attention U-Net, U-Net++, and NAU-Net, were evaluated and compared. The model complexity was evaluated by the number of floating points (FLOPs) and multiple adds (MAdds), and it was calculated with the help of Torchstat 0.0.7. Table 1 lists the total number of parameters, total memory demand, number of FLOPs, and number of MAdds. Table 1 shows that the computational cos of the U-Net family is higher than other models including FCN and SegNet. It should be also noted that FCN and SegNet usually need a relative larger number of training samples to obtain a satisfactory identification accuracy, which finally results in a significant increase of the training cost.

**Table 1:** Parameters, memory, FLOPs, and Madd of different models.

Models	Number of parameters (MB)	Total memory (GB)	FLOPs (G)	MAdds (G)
FCN	15.11	1.07	102.13	203.94
SegNet	29.44	1.14	203.02	405.73
Attention U-Net	34.88	6.06	337.26	673.77
U-Net++	36.63	8.08	698.94	1,400
NAU-Net	37.25	7.31	449.53	898.19

Moreover, Table 1 also demonstrates that the number of parameters in NAU-Net is slightly higher than those of attention U-Net and U-Net++. The total memory of NAU-Net is 20.63% higher than that of attention U-Net and 9.53% lower than that of U-Net++. Moreover, the number of FLOPs in NAU-Net is 33.29% higher than that of attention U-Net, it is 35.68% lower than U-Net++. The number of MAdds in NAU-Net is 33.31% higher than that of attention U-Net, which is 35.84% lower than that of U-Net++. To reduce the semantic gap between the low- and high-dimensional feature maps, a series of nested pathways are designed in the U-Net++, and as a result the computational cost accordingly increases. However, in the NAU-Net, only the neighboring high- and low-dimensional feature maps are linked. Moreover, since only the two feature maps with the same dimension are connected by an attention gate in the attention U-Net, the attention U-Net has less parameters than NAU-Net. Therefore, the computational cost of NAU-Net is between the cost of attention U-Net and U-Net++.

#### 4.2.2 DRIVE image segmentation

**Figure 6:**
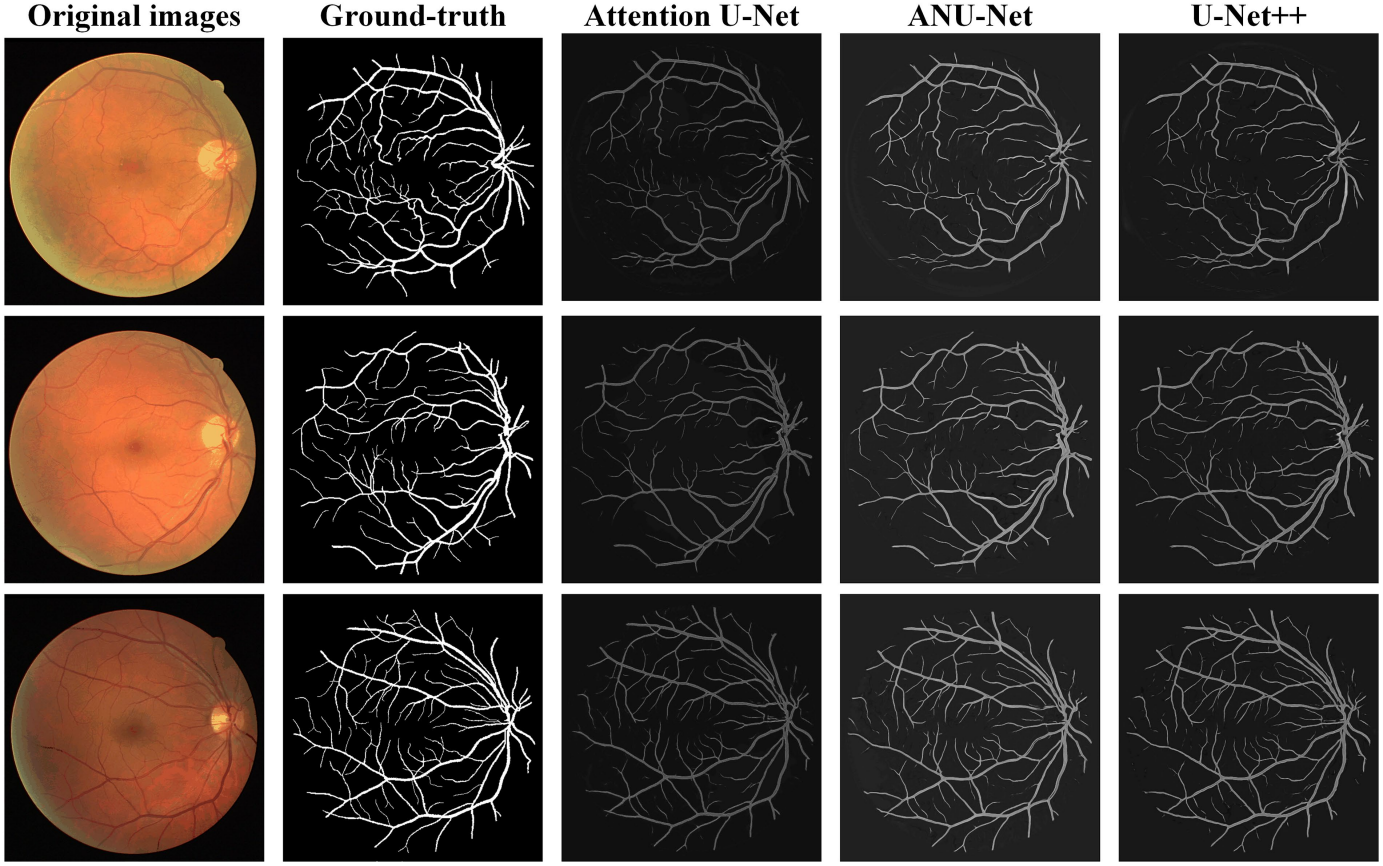
Image segmentation results of different models for DRIVE dataset.

Figure 6 shows three DR fundus images of the DRIVE dataset, their ground-truth images of retinal blood vessels, and the identification results of attention U-Net, U-Net++, and NAU-Net. Figure 6 clearly demonstrates that the proposed NAU-Net can identify some tiny and small retinal blood vessels of the DR fundus while the other two models detect less, which indicates that the fusion operation of the neighboring feature maps successfully extracts detailed features from the encoder, and therefore the proposed NAU-Net displays a better performance of tiny and small retinal blood vessel segmentation than attention U-Net and U-Net++.

Table 2 compares the segmentation performance of the DRIVE DR images obtained using the proposed NAU-Net and other 5 existing models including FCN, SegNet, attention U-Net and U-Net++. Table 2 clearly shows that the proposed NAU-Net obtained the maximum values of the Dice score, IoU, and accuracy for DR image segmentation of the DRIVE dataset among the five models. Since FCN and SegNet usually needs a relative larger number of training samples to obtain a satisfactory performance, their evaluation metrics are much lower than the models of U-Net family. Moreover, compared to attention U-Net and U-Net++, NAU-Net achieves a performance improvement from 0.18 to 7.10%, which indicates that the proposed NAU-Net has a stronger DR fundus image segmentation ability for the DRIVE dataset than the other two U-Net variants.

**Table 2:** DRIVE DR image segmentation performance of NAU-Net and other models.

Models		Metrics (Mean ± Standard deviation)			
		Dice	IoU	Accuracy	Precision
FCN		0.614 ± 0.109	0.45 ± 0.095	0.940 ± 0.008	0.704 ± 0.082
SegNet		0.663 ± 0.111	0.505 ± 0.109	0.942 ± 0.028	0.743 ± 0.148
Attention U-Net		0.730 ± 0.145	0.592 ± 0.149	0.950 ± 0.039	0.799 ± 0.16
U-Net++		0.745 ± 0.147	0.609 ± 0.134	0.960 ± 0.013	0.820 ± 0.068
NAU-Net		0.750 ± 0.133	0.613 ± 0.126	0.962 ± 0.013	0.855 ± 0.055
Improvement (%)	Over Attention U-Net	2.64	3.50	1.21	7.10
	Over U-Net++	0.69	0.71	0.18	4.29

#### 4.2.3 HRF image segmentation

**Figure 7:**
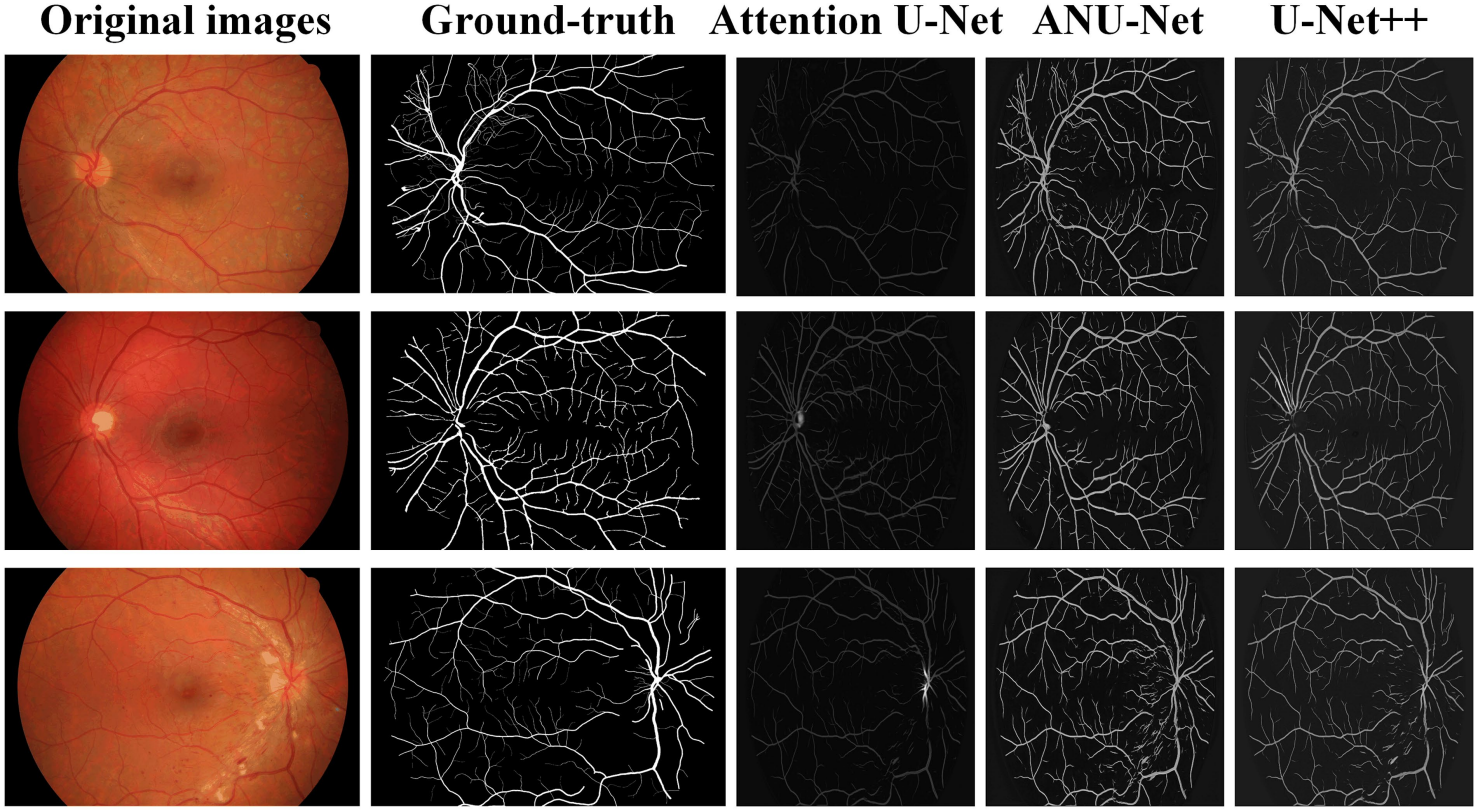
Image segmentation results of different models for HRF dataset.

Figure 7 shows three DR fundus images from the HRF dataset, their ground-truth images of retinal blood vessels, and the identification results of attention U-Net, U-Net++, and NAU-Net. Figure 7 shows that after the training, attention U-Net successfully detects most of the large vessels, while U-Net++ identifies some tiny retinal blood vessels that are not identified in the original image or ground truth. By contrast, the proposed NAU-Net correctly detects most of the vessels with the help of the fusion operation of the neighboring feature maps, including some tiny ones, which demonstrates that the proposed NAU-Net displays a better performance in retinal blood vessel segmentation than attention U-Net and U-Net++.

Table 3 compares the segmentation performances of the HRF DR images obtained using the proposed NAU-Net and other 5 existing models including FCN, SegNet, attention U-Net, and U-Net++. Similarly, Table 3 demonstrates that FCN and SegNet display a relative worse performance than the U-Net family. Table 3 also clearly shows that the proposed NAU-Net obtained the maximum values of the accuracy and precision for DR image segmentation of the HRF dataset among the attention U-Net, U-Net++, and NAU-Net, and its Dice and IoU are very close to the ones of U-Net++. Moreover, compared to attention U-Net and U-Net++, NAU-Net achieves a performance improvement from 0.28 to 9.19%, which indicates that NAU-Net has a stronger ability to DR fundus image segmentation for the HRF dataset than the other two U-Net variants, and the improvement of the proposed model is benefit to feature extraction.

**Table 3:** HRF DR image segmentation performance of NAU-Net and other models.

Models		Metrics (Mean ± Standard deviation)			
		Dice	IoU	Accuracy	Precision
FCN		0.547 ± 0.013	0.377 ± 0.012	0.924 ± 0.008	0.497 ± 0.047
SegNet		0.592 ± 0.096	0.427 ± 0.1	0.952 ± 0.003	0.78 ± 0.086
Attention U-Net		0.765 ± 0.035	0.621 ± 0.046	0.965 ± 0.003	0.76 ± 0.079
U-Net++		0.787 ± 0.044	0.651 ± 0.06	0.966 ± 0.006	0.733 ± 0.087
NAU-Net		0.786 ± 0.032	0.649 ± 0.044	0.969 ± 0.004	0.801 ± 0.108
Improvement (%)	Over Attention U-Net	2.74	4.49	0.37	5.39
	Over U-Net++	−0.09	−0.29	0.28	9.19

#### 4.2.4 CHASEDB image segmentation

**Figure 8:**
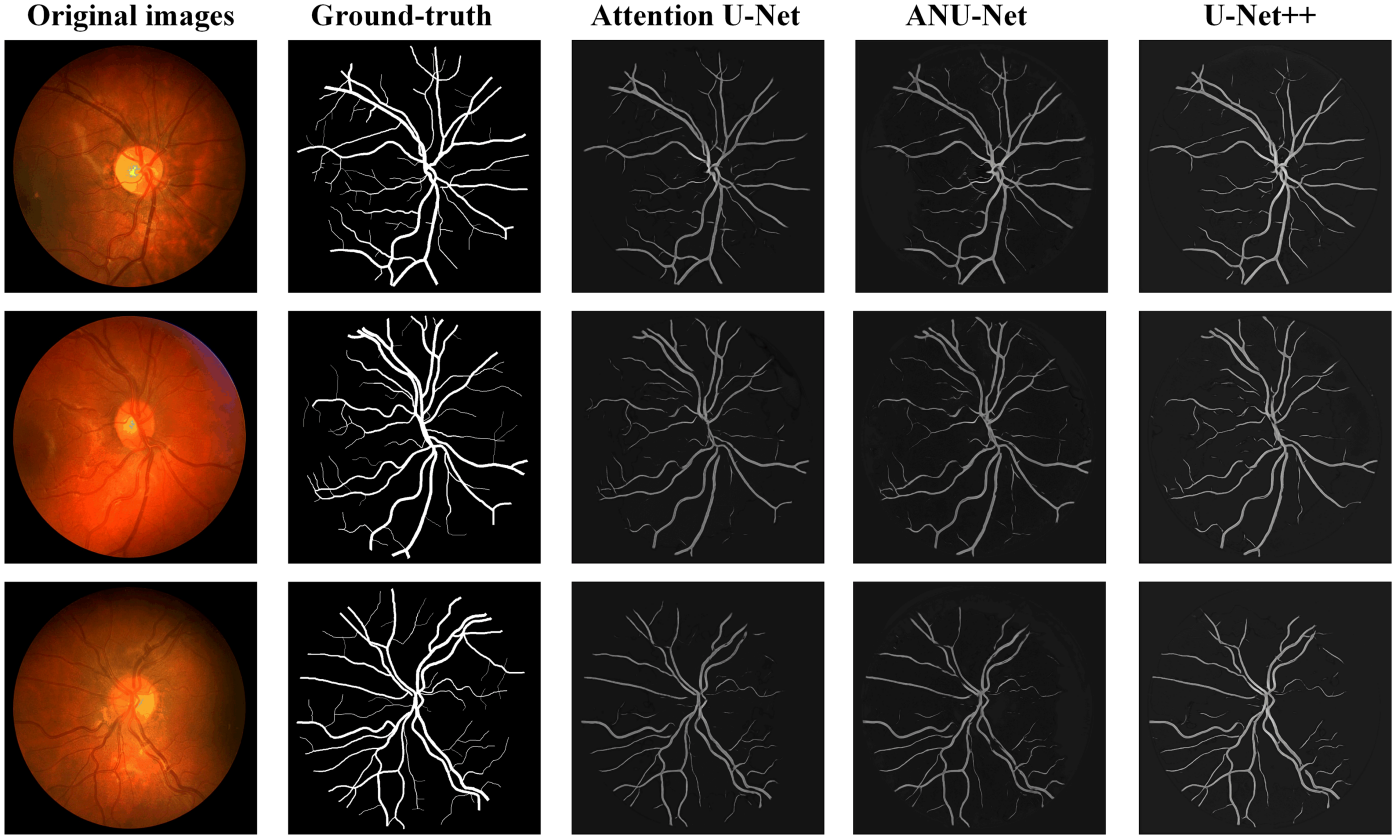
Image segmentation results of different models for CHASEDB dataset.

Figure 8 shows three DR fundus images from CHASEDB, their ground-truth images of retinal blood vessels, and the identification results of attention U-Net, U-Net++, and NAU-Net. Table 4 lists the segmentation performance for the CHASEDB DR images obtained using the proposed NAU-Net and other 5 existing models including FCN, SegNet, attention U-Net, and U-Net++. Table 4 clearly shows that the proposed NAU-Net obtains the maximum value of the Dice score, IoU, and accuracy for DR image segmentation of the CHASEDB dataset among the five models, and U-Net++ achieves the highest precision. Moreover, compared to attention U-Net and U-Net++, NAU-Net improves the segmentation performance with an average increase of 0.08 to 3.60%, which demonstrates that NAU-Net has a stronger ability for image segmentation for the CHASEDB dataset than the other two U-Net variants.

**Table 4:** CHASEDB DR image segmentation performance of NAU-Net and other models.

Models		Metrics (Mean ± Standard deviation)			
		Dice	IoU	Accuracy	Precision
FCN		0.625 ± 0.081	0.459 ± 0.082	0.944 ± 0.006	0.584 ± 0.051
SegNet		0.475 ± 0.157	0.325 ± 0.13	0.948 ± 0.004	0.765 ± 0.067
Attention U-Net		0.736 ± 0.104	0.592 ± 0.116	0.967 ± 0.006	0.800 ± 0.059
U-Net++		0.738 ± 0.085	0.591 ± 0.095	0.968 ± 0.005	0.839 ± 0.051
NAU-Net		0.755 ± 0.052	0.609 ± 0.063	0.969 ± 0.003	0.829 ± 0.058
Improvement (%)	Over Attention U-Net	2.54	2.86	0.19	3.60
	Over U-Net++	2.30	3.02	0.08	−1.18

## 5 Conclusion

In this study, to achieve a balance between identification performance and computational cost, a modified U-Net called NAU-Net is proposed for image segmentation of the DR fundus. In our new network, only the neighboring high- and low-dimensional feature maps of both the encoder and decoder are fused using four attention gates. With the help of this improvement, the common target features in the high-dimensional feature maps of encoder are enhanced, and they are also fused with the low-dimensional feature map of decoder by using these attention gates. Moreover, this network uses only neighboring layers and does not include inner layers commonly used in U-Net++. Consequently, the proposed network incurs a better identification performance with a lower computational cost. The experimental results of three open datasets of DR fundus images, including DRIVE, HRF, and CHASEDB, show that the proposed NAU-Net obtains higher scores for the Dice score, IoU, accuracy, and precision than FCN, SegNet, attention U-Net and U-Net++, while its computation cost is between the costs of the two models of attention U-Net and U-Net++. Therefore, the proposed NAU-Net exhibits better performance with a relatively low computational cost and provides an efficient novel method for DR fundus image segmentation and a new automatic tool for DR-related eye disease diagnosis. In future work, we will develop an end-to-end automatic diagnosis model that combines the proposed architecture with other classification models. Moreover, the architecture will be further improved for multitask image segmentation of DR fundus images with multiple types of lesions.

## Data availability statement

The original contributions presented in the study are included in the article/supplementary material, further inquiries can be directed to the corresponding author.

## Author contributions

TZ: Conceptualization, Formal analysis, Methodology, Software, Writing – original draft, Writing – review & editing. YG: Conceptualization, Data curation, Formal analysis, Funding acquisition, Writing – original draft, Writing – review & editing. DT: Software, Validation, Writing – original draft. LY: Formal analysis, Writing – original draft. GL: Conceptualization, Resources, Supervision, Writing – review & editing.
